# Exosomes of stem cells from human exfoliated deciduous teeth as an anti-inflammatory agent in temporomandibular joint chondrocytes via miR-100-5p/mTOR

**DOI:** 10.1186/s13287-019-1341-7

**Published:** 2019-07-29

**Authors:** Ping Luo, Chao Jiang, Ping Ji, Menghong Wang, Jie Xu

**Affiliations:** 1 College of Stomatology, Chongqing Medical University, Chongqing, China; 2 Chongqing Key Laboratory for Oral Diseases and Biomedical Sciences, Chongqing, China; 3 Chongqing Municipal Key Laboratory of Oral Biomedical Engineering of Higher Education, Chongqing, China; 4 Department of Oral and Maxillofacial Surgery, The Affiliated Hospital of Stomatology, Chongqing Medical University, No. 426, North Songshi Road, Yubei District, Chongqing, 401147 China; 5 Pediatric Dentistry Department, The Affiliated Hospital of Stomatology, Chongqing Medical University, No. 426, North Songshi Road, Yubei District, Chongqing, 401147 China

**Keywords:** Osteoarthritis, SHED, Exosomal microRNA, Inflammation, Temporomandibular joint

## Abstract

**Objectives:**

Temporomandibular joint osteoarthritis (TMJOA) is an inflammatory joint disease. This study investigated whether exosomes (Exos) of stem cells from human exfoliated deciduous teeth (SHEDs) have a therapeutic effect on TMJ inflammation and elucidated the underlying mechanisms.

**Materials and methods:**

SHEDs were verified by flow cytometry. SHED-Exos were identified by western blotting, nanoparticle tracking analysis, and transmission electron microscopy. Western blot and RT-qPCR were performed to verify the anti-inflammatory effects of SHED-Exos. MicroRNA (miRNA) array analysis was conducted to determine the miRNA expression profiles of SHED-Exos, and the key pathways were analyzed. After chondrocytes were treated with an miR-100-5p mimic or rapamycin, relative expression of genes was measured by RT-qPCR and western blotting. A luciferase reporter assay was performed to reveal the molecular role of the exosomal miR-100 target, mTOR.

**Results:**

MiR-100-5p was enriched in the SHED-Exos. Treatment with SHED-Exos suppressed the expression of interleukin-6 (IL-6), IL-8, matrix metalloproteinase 1 (MMP1), MMP3, MMP9, MMP13, and disintegrin and metalloproteinase with thrombospondin motifs 5 (ADAMTS5). Chondrocytes treated with the miR-100 mimic showed lower expression of MMP1, MMP9, MMP13, ADAMTS5, and mTOR. In contrast, miR-100 downregulation upregulated the MMPs and mTOR. Rapamycin treatment upregulated miR-100 and downregulated MMPs and ADAMTS5. Furthermore, the luciferase reporter assay demonstrated that miR-100-5p directly targeted the *mTOR* 3′ untranslated region and that SHED-Exos miR-100-5p repressed mTOR expression.

**Conclusions:**

This study demonstrated that SHED-Exos suppress inflammation in TMJ chondrocytes and may thus be a novel therapeutic agent for TMJ inflammation.

## Introduction

The temporomandibular joint (TMJ) is a synovial joint that performs the most complicated movement in the human body. Osteoarthritis (OA) is a degenerative disease characterized by progressive cartilage degradation, subchondral bone remodeling, synovitis, and chronic pain [1]. Recent studies showed that inflammation is strongly involved in the pathogenesis of TMJOA, which leads to high expression of inflammatory cytokines, including interleukin-6 (IL-6) and IL-8 [2]. Increased expression of inflammatory factors causes pathological changes in chondrocytes, thereby upregulating catabolic enzymes in the cartilage matrix. Excess production of matrix-degrading proteases including matrix metalloproteinases (MMPs) and a disintegrin and metalloproteinase with thrombospondin motifs (ADAMTSs), results from disruption of metabolic homeostasis and leads to cartilage degradation with inflammation [3, 4]. IL-1β, a major proinflammatory cytokine, is implicated in the destructive effects characterized by increasing cartilage degradation and suppression of cartilage matrix synthesis [5, 6]. There are several current treatment strategies for relieving inflammation and preventing the degradation of the joint complex. However, no effective strategy exists to repair and regenerate the damaged TMJ.

Lately, there has been an increasing interest in the use of mesenchymal stem cells (MSCs) for the treatment of TMJOA [7]. Stem cells from human exfoliated deciduous teeth (SHEDs) are an ideal cell source for regenerative medicine as they are highly proliferative, have high multipotency, and are immunosuppressive [8–11]. It is well-documented that MSCs release a rich secretome containing large amounts of cytokines, growth factors, chemokines, and extracellular nanoparticles. The most important nanoparticles shed by MSCs are exosomes [12, 13]. Recently, increasing evidence has demonstrated that the small membrane vesicles called exosomes play a key role in the mechanism of action in stem cell therapy [12, 14]. Exosomes are small membrane-bound vesicles (30–100 nm in diameter) that are released into the extracellular space by most cell types under both physiological and pathological conditions [15, 16]. Exosomes contain microRNAs (miRNAs), mRNAs, DNA, and signaling proteins and can modulate the activities of recipient cells [12]. Exosomes have been identified as the principal agent mediating the therapeutic efficacy of SHEDs in several medical conditions, such as traumatic brain injury [17], wound healing [18], and acute inflammation [19]. Moreover, it is believed that exosomes hold great promise as a cell-free therapy because of their safety and low immunogenicity [20].

Exosome-borne miRNAs have been proposed as a means of intercellular communication. MiRNAs are small 18–24 nucleotide-long noncoding RNAs that control gene expression post-transcriptionally [21]. MiRNAs play a key part in cellular proliferation, development, and tissue remodeling [22]. However, it is still unknown which miRNAs are expressed in SHED exosomes (SHED-Exos) and how they are regulated.

Therefore, we hypothesized that SHED-Exos could be beneficial and serve as a promising treatment for TMJOA. In this study, we aimed to explore the therapeutic potential of SHED-Exos and their mechanisms of action in TMJOA. This study may thus help develop a prophylactic agent for TMJOA.

## Materials and methods

### Ethics statement for human samples

Ethics approval was obtained from the Committee of Chongqing Medical University, and written informed consent was obtained from all the participants. Collection and processing of condylar and human exfoliated deciduous teeth was conducted in accordance with the Declaration of Helsinki.

### Cell culture

SHEDs were purchased from China Oral Stem Cell Bank (Beijing, China) and originated from nine normal human deciduous incisors collected from 5- to 8-year-old individuals. SHEDs were plated in T175 culture flasks in α-MEM supplemented with 10% fetal bovine serum (FBS) and were incubated at 37 °C in an atmosphere containing 5% CO_2_ at 100% humidity. To obtain chondrocytes, human condyles were collected from five patients with condylar fracture during the surgical procedure of mandibular condylectomy. Cartilage were immediately isolated from the TMJ cartilage layer with a surgical blade, and carefulness was taken not to disturb the subcondral bone. All cartilage specimens were washed three times with phosphate-buffered saline (PBS) to remove blood prior to mincing and digestion for 30 min in 0.25% trypsin (HyClone, USA) followed by digestion with 3 mg/ml type I collagenase for 1 h. The chondrocytes were washed, resuspended in DMEM (HyClone, USA) supplemented with 10% FBS (HyClone, USA), 1% penicillin/streptomycin solution, and cultured at 37 °C and 5% CO_2_. The chondrocytes were then cultured in dishes at a density of 1 × 10^5^ cells/cm^2^. The cells used in the experiments were at passage 4. For gene and protein expression experiments, chondrocytes were seeded at 1 × 10^5^ cells/cm^2^ in a 6-well culture plate. The chondrocytes were then pretreated with SHED conditioned medium (SHED-CM) or SHED-Exos (150 μl per well, equivalent to exosomes from 1.5 × 10^7^ cells) for 2 h followed by stimulation with the proinflammatory factor IL-1β (10 ng/ml). For gene expression analysis, samples were harvested after 24 h, whereas for protein expression analysis, samples were harvested at 48 h.

### Isolation of exosomes

SHEDs were seeded and grown to confluence in a T175 culture flask. Exosomes were isolated from the culture medium of cells containing exosome-free FBS. One day prior to isolation, the cells were washed with serum-free medium and cultured for 48 h. Exosomes were isolated in accordance with previously published protocols [23]. The exosome purification procedure was based on differential ultracentrifugation. All centrifugation steps were performed at 4 °C. First, to eliminate dead cells and large cell debris, culture supernatants collected from SHEDs were centrifuged successively at increasing speeds (300×*g* for 10 min, 2000×*g* for 10 min, and 20,000×*g* for 30 min). The resultant supernatants were ultracentrifuged at 100,000×*g* for 70 min in an ultracentrifuge (Hitachi, CP100NX, Japan). Finally, the pellets were washed with 10 ml of PBS and centrifuged one last time at 100,000×*g* for 70 min to eliminate contaminating proteins. Final pellets of the exosomal fraction were resuspended in sterile 100 μl PBS and stored at − 80 °C. Exosome suspensions were normalized to the cell number and diluted to ensure that 100 μl of suspension contained exosomes isolated from 1 × 10^7^ cells.

### SHED-CM

The conditioned medium (CM) from SHEDs was generated as follows: SHEDs (passage 3–5) were seeded in a T175 cell culture flask for 24 h at 37 °C with 5% CO_2_. Next, the culture medium was removed from each flask, and the flasks were washed twice with PBS to remove cell debris. The SHEDs were then cultured in serum-free medium for 48 h. The SHED-CM was collected and centrifuged at 1500 rpm for 5 min, followed by centrifugation at 3000 rpm for 5 min to remove cell debris.

### Identification of SHEDs

SHEDs (passage 3) were verified using flow cytometry to assess the phenotypic characteristics. CD73 ((BD, USA), CD146 ((BD, USA), and CD34 ((BD, USA) were used for detection. To identify the multiple differentiation potential of SHEDs, cells were induced to differentiate in osteogenic and adipogenic medium for 3 weeks. Flow cytometry and differentiation protocols for SHEDs were performed as described previously [24]. Alizarin Red (Solarbio, China) and Oil Red O (Solarbio, China) were used to identify these cell types, respectively.

### Transmission electron microscopy (TEM)

TEM was performed to verify the presence of exosomes in purified samples. The enriched exosomes were placed onto formvar/carbon-coated nickel TEM grids and incubated for 30 min. With the excess fluid removed, the samples were then stained with 3% phosphotungstic acid (pH 6.9) for 5 min and then subjected to TEM.

### Nanoparticle tracking analysis (NTA)

A Nanosight Tracking Analysis system (Brookhaven Instruments Corp, USA) was employed to determine the particle size and particle concentration per milliliter.

### Fluorescent labeling of exosomes: endocytosis experiments

The exosomes were labeled with the PKH-67 Labeling Kit (Sigma, USA) according to the manufacturer’s protocol. A control reaction was performed with PBS instead of exosomes during each labeling reaction, which served as a control in subsequent experiments to account for nonspecific staining resulting from the labeling procedure. SHEDs were seeded in 6-well culture plates. At 24 h after cell seeding, the labeled exosomes or a control mixture were added to the culture medium and incubated for 30 min, 1 h, or 2 h at 37 °C. The coverslips were washed thrice with PBS and fixed in 4% neutral buffered formalin solution, and the nuclei were stained with 4′,6-diamidine-2′-phenylindole (DAPI) according to previously published protocols [25]. Then, images were captured using an Olympus microscope (Olympus, Japan).

### Firefly luciferase and *Renilla* luciferase assays

293T cells were seeded in a 96-well plate, cultured to 70% confluence, and transfected with either the h-mTOR-3′ UTR plasmid or hsa-miR-100-5p/Negative Control (Hanbio Biotechnology, China) using a transfection reagent (Hanbio Biotechnology, China). The cells were collected at 48 h after transfection. Luciferase activity was determined in cell lysates with a Dual-Luciferase Reporter Assay System (Promega, USA) according to the manufacturer’s instructions. Firefly luciferase and *Renilla* luciferase activities were detected on a Veritas Microplate Luminometer (Promega, USA). The firefly luciferase to *Renilla* luciferase ratio was calculated for each sample and was normalized to the ratio for NG-cultured cells.

### Reverse transcription-quantitative PCR (RT-qPCR) for mRNA and miRNA expression analyses

For gene expression analysis, cells were collected, washed thrice with PBS, and lysed in RNAiso plus (Takara, Japan). Total RNA was isolated and reverse-transcribed using the PrimeScript™ RT Reagent Kit with gDNA Eraser (Takara, Japan) to obtain cDNA. For miR-100 expression analysis, after RNA isolation, first-strand cDNA was synthesized via reverse transcription using the Mqklir-X™ miRNA First Strand Synthesis Kit (Takara, Japan) according to the manufacturer’s instructions. qPCR was then carried out on a Bio-Rad real-time PCR system (CFXConnect, USA) for 40 cycles with the Power TB Green PCR Master Mix (Takara, Japan). The expression levels of target genes were normalized to the control housekeeping gene *GAPDH.* MiR-100 expression was normalized to that of *U6*. Gene expression data were analyzed by the 2^−ΔΔCt^ method. For primer sequences, refer to Table 1.

**Table 1:** Primer sequences for RT-qPCR

Gene	Forward primer (5′–3′)	Reverse primer (5′–3′)
IL-6	AGCCCACCGGGAACGA	GGACCGAAGGCGCTTGT
IL-8	AGAAGTTTTTGAAGAGGGCTGAGA	AGTTTCACTGGCATCTTCACTGATT
MMP1	ACTGCCAAATGGGCTTGAAG	TTCCCTTTGAAAAACCGGACTT
MMP3	GAGGCATCCACACCCTAGGTT	TCAGAAATGGCTGCATCGATT
MMP9	CCCTTGTGCTCTTCCCTGGA	TCTGCCACCCGAGTGTAACC
MMP13	ATTAAGGAGCATGGCGACTTCT	CCCAGGAGGAAAAGCATGAG
ADAMTS5	GGCCTCCATCGCCAATAGG	GGATAGCTGCATCGTAGTGCT
miR-100	GTGTTCAAGCCTAGATGCCCAA	GCATCTAGGCTTGAACACGCC
mTOR	TCTTTCATTGGAGACGGTTTGG	AGGTTTTCATGGGATGTCGCT
U6	CGCTTCGGCAGCACATATAC	AAATATGGAACGCTTCACGA
GAPDH	CTTTGGTATCGTGGAAGGACTC	GTAGAGGCAGGGATGATGTTCT

*IL-6* Interleukin-6, *IL-8* Interleukin-8, *MMP1* matrix metalloproteinase-1, *MMP3* matrix metalloproteinase-3, *MMP-9* matrix metalloproteinase-9, *MMP13* matrix metalloproteinase-13, *ADAMTS5* disintegrin and metalloproteinase with thrombospondin motifs 5, *miR-100* microRNA-100, *mTOR* mammalian target of rapamycin, *GAPDH* glyceraldehyde 3 phosphate dehydrogenase, *RT-qPCR* Reverse transcription-quantitative polymerase chain reaction

### Western blotting

Chondrocytes were pretreated with SHED-Exos or SHED-CM and then incubated with or without 10 ng/mL IL-1β for 48 h before the cell lysates were prepared. For preparation of SHED-Exos, a purified exosome pellet was resuspended in RIPA Lysis Buffer (Beyotime, China). To prepare total-cell lysates, cells were washed twice with cold PBS and lysed in RIPA Lysis Buffer (Beyotime, China) for 15 min on ice. Next, the exosomes were pelleted and total protein concentration was quantified using the Enhanced BCA Protein Assay Kit (Beyotime, China). For western blot analysis, 30 μg of cell lysates or SHED-Exo lysates were loaded for each sample and separated by 10% SDS-PAGE. The proteins were transferred to polyvinylidene difluoride membranes and then blotted with antibodies against calnexin (1:1000, CST, USA), CD9 (1:800, ProteinTech, USA), CD63 (1:1000, Abcam, USA), TSG101 (1:800, ProteinTech, USA), MMP1 (1:1000, Bioss, China), MMP9 (1:800, Zen Bioscience, China), and mTOR (1:500, CST, USA). GAPDH (1:2000, Bioss, China) was blotted as the loading control. Images were captured and analyzed with a computer program (ImageJ, USA). The presented blots are representative of three separate reproducible experiments.

### Bioinformatics analysis

The sequences that matched the human genome were chosen for subsequent analysis. miRNA identification was performed in the miRBase database, and the miRNA expression levels were normalized to transcripts per million clean tags. Microarray analyses were performed on the miRNA using an Affymetrix GeneChip® miRNA 4.0 Array at the Integrated Genomics Core, Augusta University, GA. Kyoto Encyclopedia of Genes and Genomes (KEGG) pathway analysis was then performed in KOBAS3.0 (http://kobas.cbi.pku.edu.cn/) to determine the participation of co-expressed genes in different biological pathways. TargetScan (http://www.targetscan.org) and miRanda (http://www.microrna.org) were employed to determine the potential miR-100-5p-binding site.

### Transfection of miRNA-100

Chondrocytes were seeded on 6-well culture slides and grown to 70% confluence. The cells were transfected with 50 nM miR-100 mimic, 100 nM miR-100 inhibitor, or the corresponding control oligonucleotide (miR-100-mimic-NC or miR-100-inhibitor-NC) using the GP-siRNA-mate-plus transfection reagent (GenePharma, China) in 2 ml of α-MEM according to the manufacturer’s instructions. Six h post-incubation, the medium was removed, and cells were washed thrice with PBS and transfected for 48 h in standard conditions. The sequences were as follows: hsa-miR-100-5p mimic (sense, 5′-AACCCGUAGAUCCGAACUUGUG-3′; antisense, 5′-CACAAGUUCGGAUCUACGGGUU-3′), hsa-miR-100-5p inhibitor (sense, 5′-CACAAGUUCGGAUCUACGGGUU-3′), mimic negative control (sense, 5′-UUUGUACUACACAAAAGUACUG-3′; antisense, 5′-CAGUACUUUUGUGUAGUACAAA-3′), and inhibitor negative control (sense, 5′-CAGUACUUUUGUGUAGUACAAA-3′). All the oligos were synthesized by RiboBio (Guangzhou, China).

### Treatment with an mTOR inhibitor

Chondrocytes were incubated with an mTOR inhibitor: rapamycin (10 nM) (Selleck, USA), for 24 h, and then stimulated with IL-1β for 24 h followed by mRNA expression analysis.

### Statistical analysis

All procedures were repeated at three biological replicates to verify the results. GraphPad Prism 7.0 (GraphPad Software, USA) was employed to perform statistical analysis. Data are presented as mean ± SEM. Results were statistically analyzed with Student’s *t* test for two-group comparisons and using one-way ANOVA with Tukey’s post hoc test for multigroup comparisons. Ns indicates no significant difference. Data with *P* values < 0.05 were considered significant. *P* < 0.05, *P* < 0.01, *P* < 0.001, and *P* < 0.0001 in figures indicate statistically significant differences and are represented as *, **, ***, and ****, respectively.

## Results

### Characterization of SHED and their differentiation potential

**Fig. 1:**
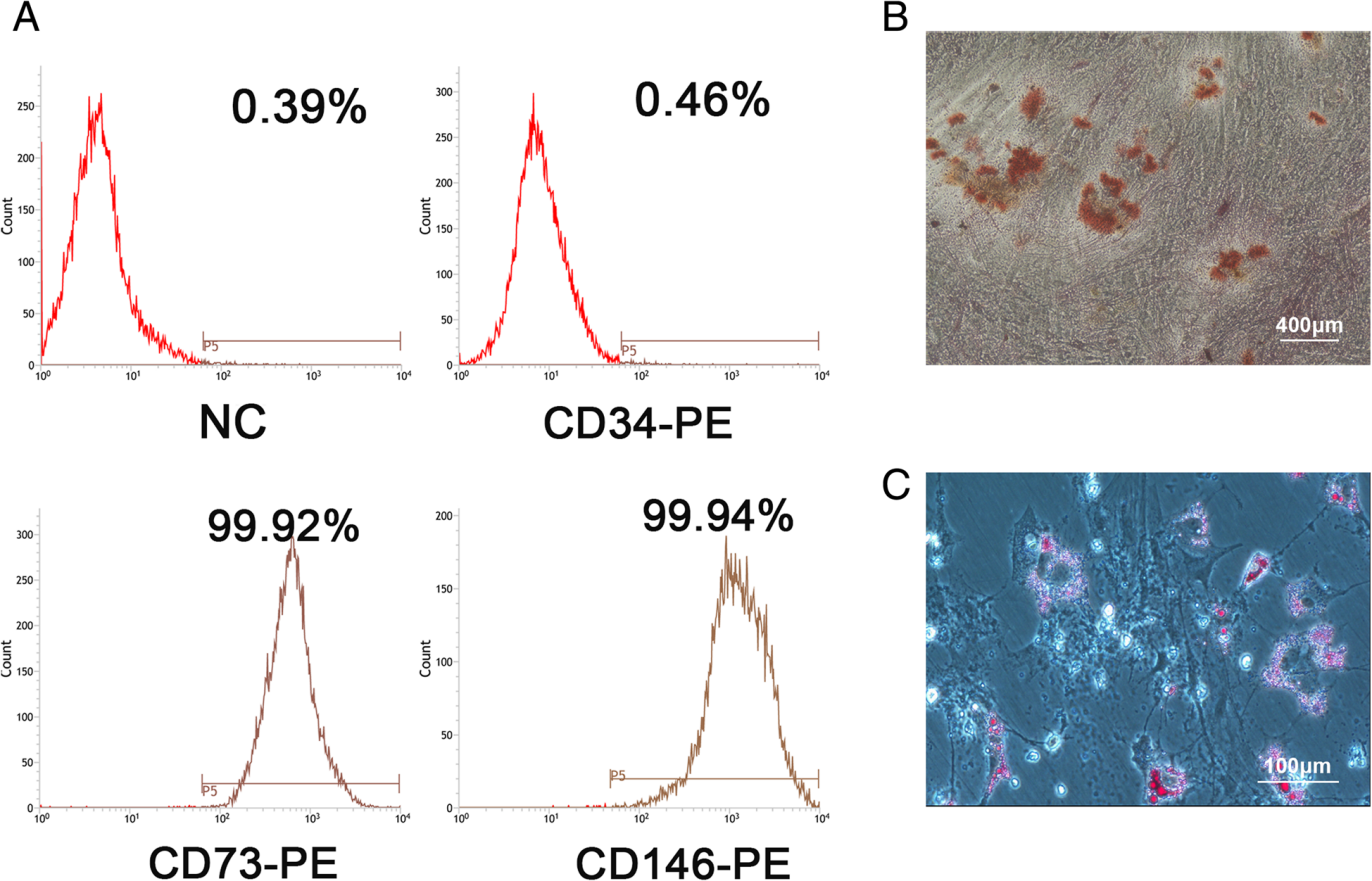
Identification of the differentiation ability and purity of SHEDs. **a** Flow cytometric analysis of SHED surface markers. **b** Alizarin red staining. Calcium salt deposition was observed (red) after 3 weeks of osteogenesis induction. **c** Oil red O staining. Fat bubbles (orange red) formed after 3 weeks of adipogenesis

SHEDs were characterized by flow cytometry after staining surface markers. These cells were found to be positive for CD73 and CD146, but did not express the hematopoietic marker CD34 (Fig. 1a). Trilineage differentiation potential was evaluated by staining with Alizarin red for osteogenic (Fig. 1b) and Oil red O for adipocytic (Fig. 1c).

### SHED-Exo identification

**Fig. 2:**
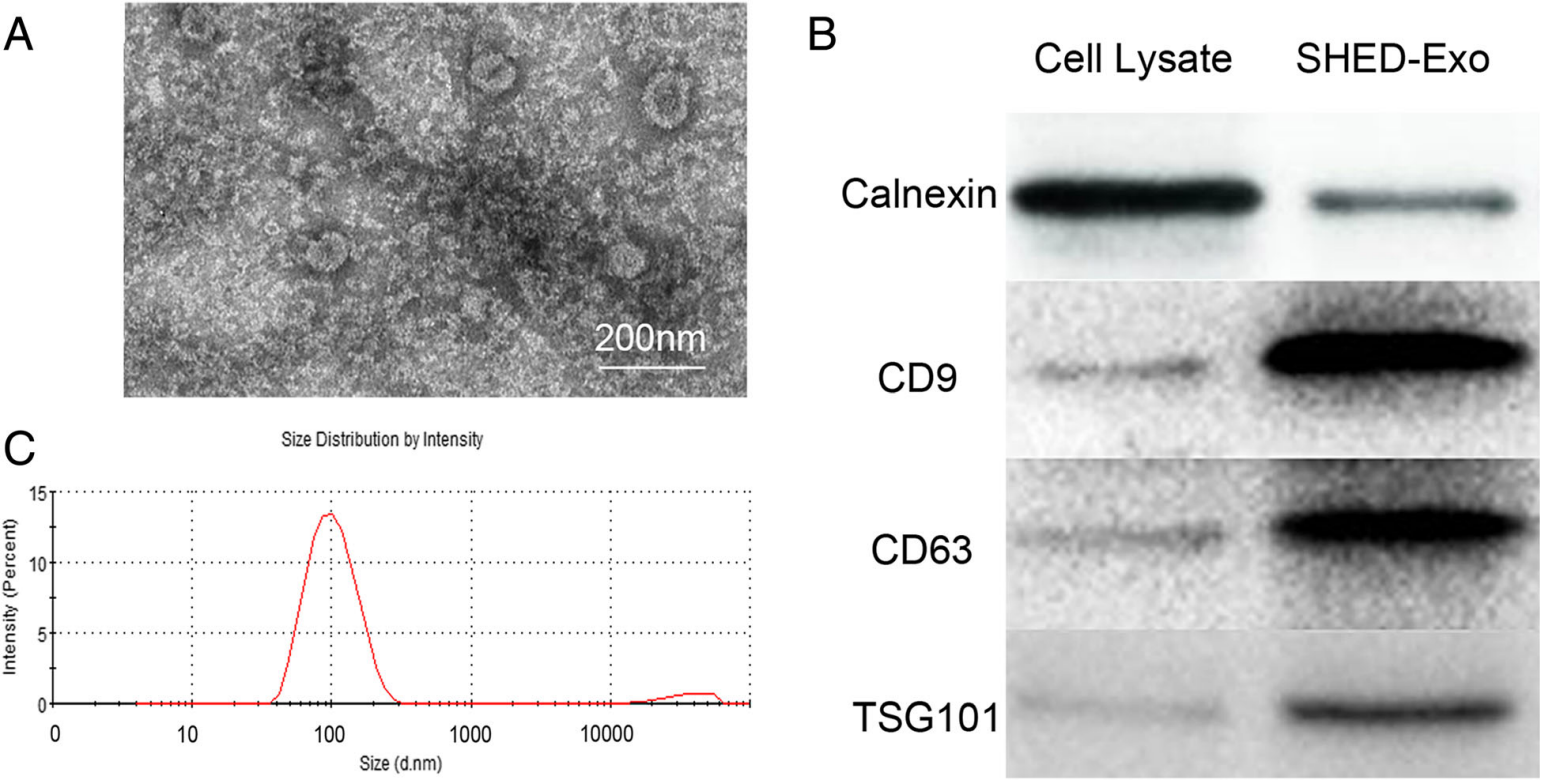
Identification of SHED-Exos. **a** Morphology of the exosomes according to TEM. The exosome vesicles were with diameters ranging from 30 to 100 nm. Scale bars indicate 200 nm. **b** Surface exosomal markers of SHED-Exos (CD63, TSG101, and CD9) were quantified by western blotting. **c** Determination of the concentration and particle size of SHED-Exos using a Nanosight Tracking Analysis system

To verify the presence of SHED-Exos in the isolates, TEM, western blotting, and NTA were performed. The electron micrographs showed that most of the exosomes had a characteristic morphology with sizes ranging from 30 to 100 nm and appeared as circular particles (Fig. 2a). Then, the expression of surface exosomal markers CD9, CD63, and TSG101 was verified by western blotting. The results indicated that SHED-Exos carried large amounts of CD9, CD63, and TSG101 compared to SHED lysates. Besides, the data showed no expression of the endoplasmic reticulum chaperone protein calnexin in SHED-Exos, suggesting that the exosomes extracted from the conditioned medium (CM) contained no cellular components. (Fig. 2b). NTA revealed that the size distribution of Exos was near 100 nm, in agreement with the characteristics of exosomes (30–100 nm; Fig. 2c). Overall, we successfully obtained exosome particles from SHEDs.

### SHED-Exos suppress proinflammatory cytokine expression in chondrocytes

**Fig. 3:**
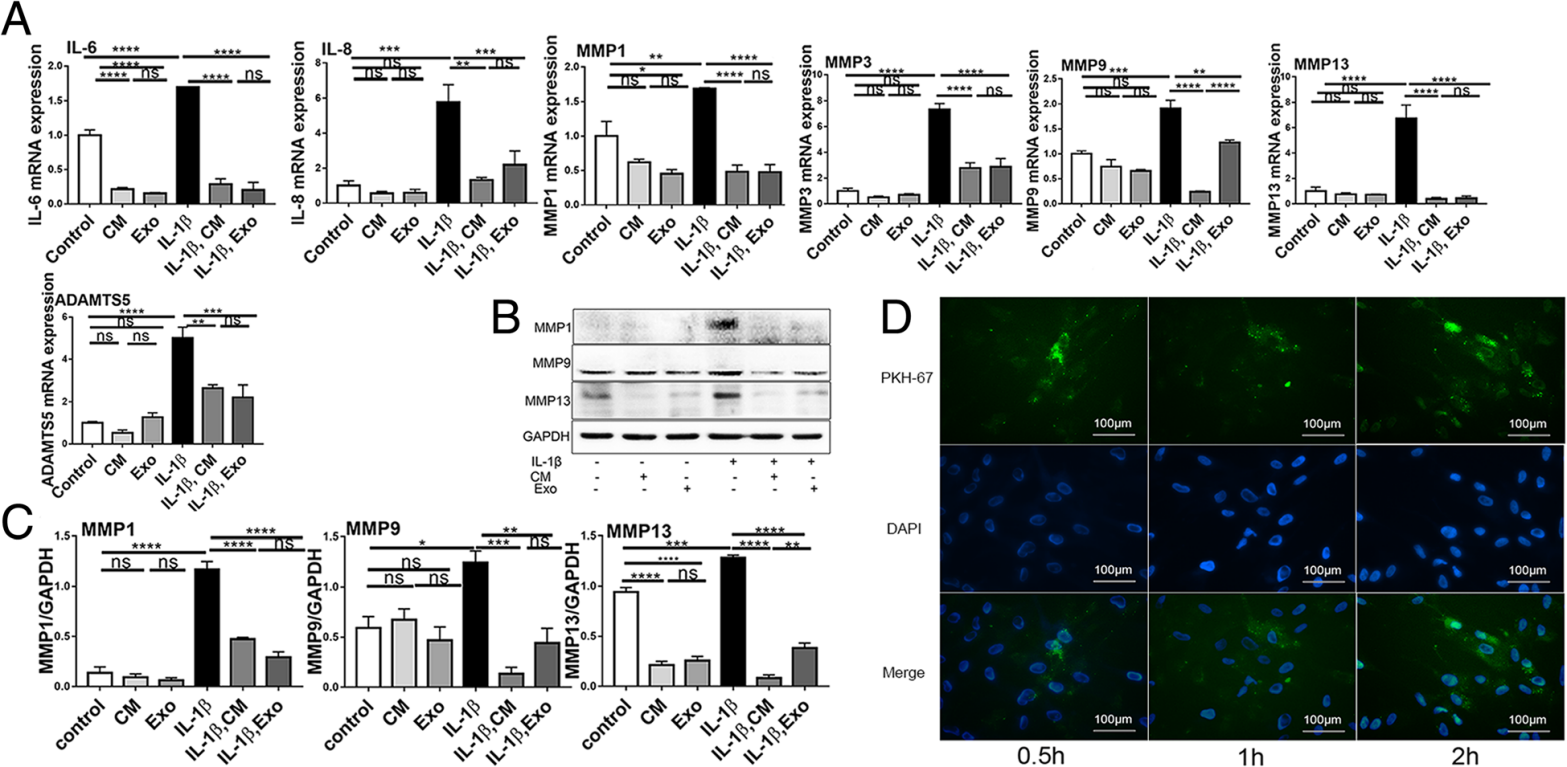
SHED-Exos suppress the expression of proinflammatory cytokines in chondrocytes. **a** Human chondrocytes were pretreated with SHED-CM or SHED-Exos followed by stimulation with IL-1β (10 ng/ml). Quantification of expression levels by RT-qPCR revealed significant downregulation of inflammatory factors after 24 h of stimulation (*n* = 3). **b** Protein levels were determined by western blotting (*n* = 3). **c** Protein semi-quantitative analysis of MMP1, MMP9, and MMP13. **d** Representative image of immunofluorescence staining shows labeled SHED-Exos (green) endocytosed by SHEDs at 37 °C (counterstaining with DAPI; blue). Data are presented as mean ± SEM. Data with *P* values < 0.05 (one-way ANOVA followed by Tukey’s test) were considered significant; *P < 0.05, **P < 0.01, ***P < 0.001, ****P < 0.001

To investigate the anti-inflammatory effects of SHED-Exos, chondrocytes were pretreated with SHED-Exos or SHED-CM for 2 h, followed by IL-1β stimulation. RT-qPCR analyses revealed that the expression levels of proinflammatory cytokines IL-6 and IL-8, and the proteases MMP1, MMP3, MMP9, MMP13, and ADAMTS5 were suppressed in the SHED-Exo-treated group and SHED-CM-treated group (Fig. 3a). Further, the results of protein analyses were consistent with these results, i.e., MMP1, MMP9, and MMP13 were downregulated in the SHED-Exo-treated cells and SHED-CM-treated cells (Fig. 3b, c). To examine whether the exosomes released by SHED were translocated into chondrocytes, endocytosis of exosomes by SHEDs was tested. The cultures were incubated with labeled exosomes at 37 °C for 30 min, 1 h, and 2 h. The isolated exosomes were labeled fluorescently (green), and chondrocyte nuclei were stained blue. Figure 3d reveals that the SHED-Exos were endocytosed by chondrocytes at 30 min, 1 h, and 2 h and that the green fluorescence increased with incubation time.

### SHED-Exos ameliorate inflammation through miR-100

**Fig. 4:**
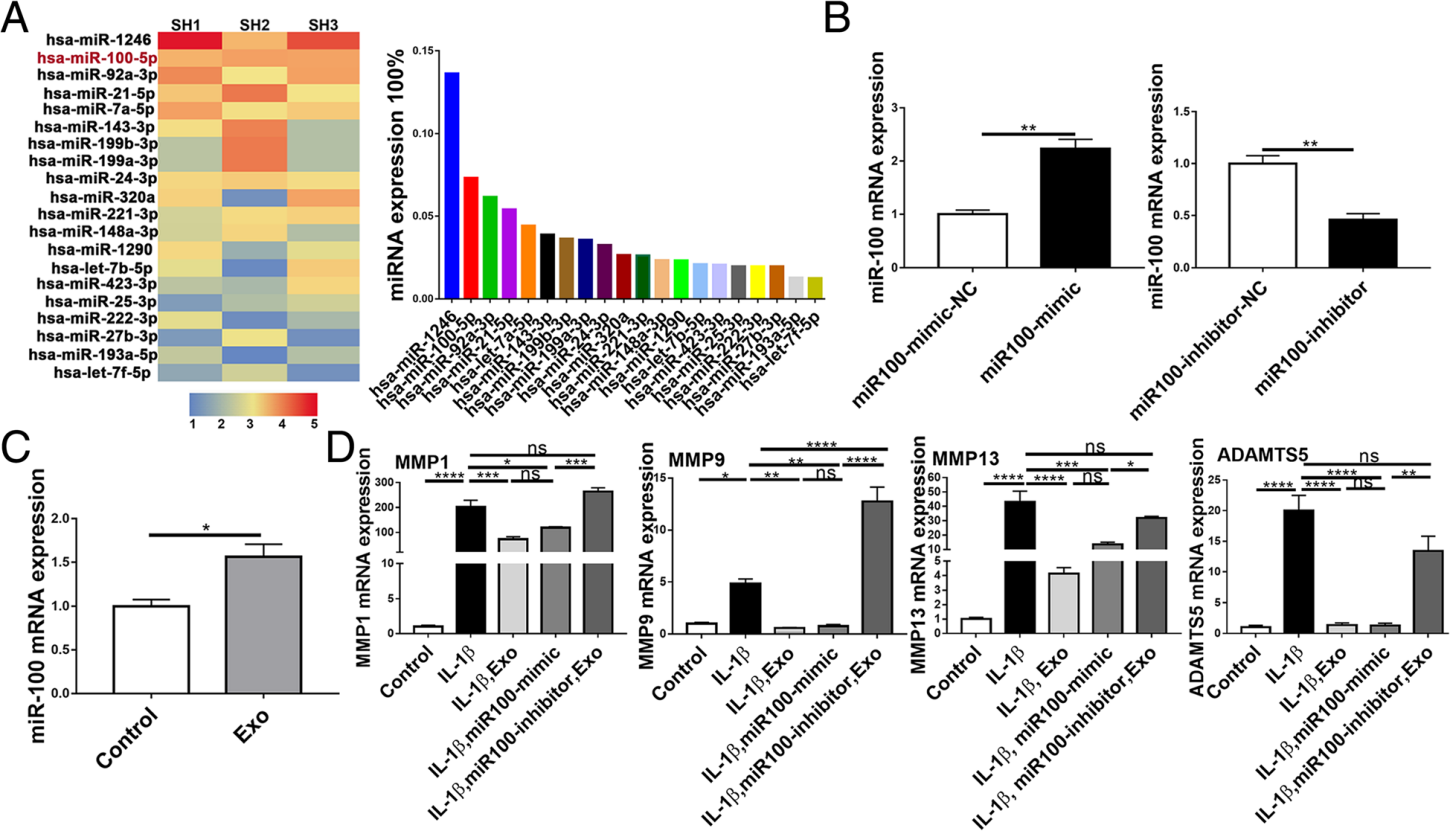
MiR-100 as a SHED-Exo-enriched miRNA that attenuates inflammation. **a** Top 20 differentially expressed miRNAs. Heatmap of exosomal miRNA expression profiles assessed by miRNA microarray analysis. Each row represents a miRNA and each column represents a sample. The color scale shown in the map indicates the expression levels of miRNAs across all samples: red shades represent high expression levels and blue shades represent lower expression levels. **b** Chondrocytes were transfected with the miR-100 mimic, miR-100 inhibitor, miR-100 mimic negative control, or miR-100 inhibitor negative control. Next, miR-100 levels were determined by RT-qPCR. Statistical analysis was performed using Student’s *t* test. **c** MiR-100 expression in SHED-Exo-treated chondrocytes was detected by RT-qPCR. Statistical analysis was performed using Student’s *t* test. **d** mRNA expression of MMP1, MMP9, MMP13, and ADAMTS5 was measured by RT-qPCR. Statistical analysis was performed using one-way ANOVA followed by Tukey’s test. Data with *P* values < 0.05 were considered significant; *P < 0.05, **P < 0.01, ***P < 0.001, ****P < 0.001, *n* = 3. All data are expressed as the mean ± SEM

The expression profile of miRNAs in SHED-Exos was determined by miRNA microarray analysis. The results uncovered the top 20 most significant differentially expressed exosomal miRNAs. MiR-100 was more abundant in SHED-Exos (Fig. 4a). To determine whether exosomal miR-100-5p from SHEDs ameliorates inflammation, miR-100-5p was either overexpressed or downregulated in chondrocytes (Fig. 4b). Figure 4c shows that miR-100 was upregulated after treatment with SHED-Exos. Furthermore, the mRNA expression levels of MMP1, MMP9, MMP13, and ADAMTS5 were significantly decreased in SHED-Exos-treated chondrocytes and in miR-100-overexpressing chondrocytes, whereas in the miR-100 inhibitor group, these proinflammatory enzymes were found to be upregulated (Fig. 4d).

### MiR-100 suppressed inflammation via repression of mammalian target of rapamycin (mTOR)

We next clarified the molecular mechanisms underlying miR-100 regulation. The bioinformatics software TargetScan (http://www.targetscan.org) and miRanda (http://www.microrna.org) revealed that *mTOR* mRNA contains a potential miR-100-5p-binding site.

**Fig. 5:**
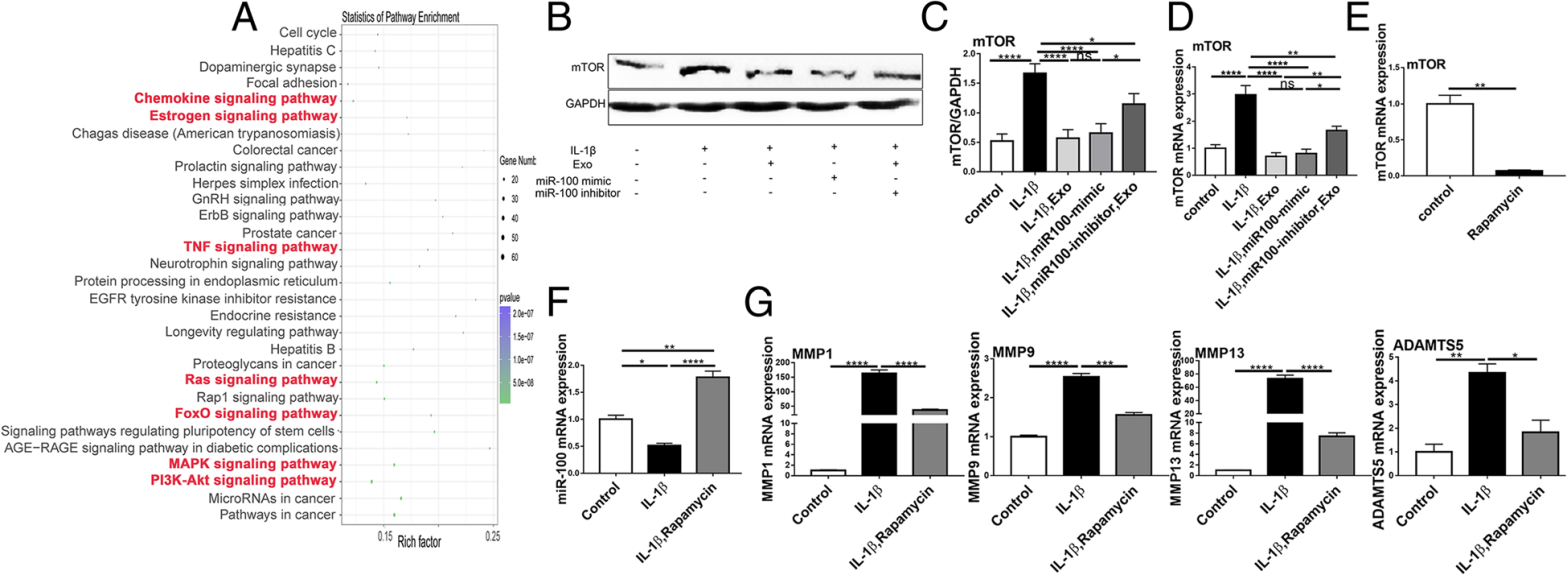
MiR-100 suppressed inflammation via repression of mammalian target of rapamycin (mTOR). **a** KEGG pathway enrichment analysis for SHED-Exos. The *X*-axis represents the percentage of the total number of annotated miRNAs in this pathway, whereas the *Y*-axis indicates the name of a pathway. **b** mTOR protein expression was analyzed by western blotting. **c** Protein semi-quantitative analysis of mTOR. **d** mTOR mRNA expression was analyzed by RT-qPCR. Statistical analysis was performed by one-way ANOVA followed by Tukey’s test. **e** Chondrocytes were treated with rapamycin (10 nM) to inhibit mTOR. Statistical analysis was performed by Student’s *t* test. **f** MiR-100 expression levels were quantified by RT-qPCR. **g** The expression of inflammatory factors was quantified by RT-qPCR. Data are presented as mean ± SEM. Data with *P* values < 0.05 were considered significant; *P < 0.05, **P < 0.01, ***P < 0.001, ****P < 0.001

Functional enrichment analysis revealed that the genes in the network were significantly involved in some KEGG pathways, such as the mTOR signaling pathway (Fig. 5a). As presented in Fig. 5b–d, RT-qPCR and western blotting both revealed that mTOR was upregulated by IL-1β and downregulated by SHED-Exos. Overexpression of miR-100 downregulated mTOR, whereas miR-100 inhibition upregulated mTOR expression. Furthermore, to investigate the role of mTOR in TMJOA-associated inflammation, rapamycin was used to inhibit mTOR expression (Fig. 5e). As depicted in Fig. 5f, mTOR inhibition induced upregulation of miR-100. The function of mTOR was also verified as treatment with the direct inhibitor of mTOR (rapamycin) suppressed the expression of MMP1, MMP9, MMP13, and ADAMTS5 (Fig. 5g).

### mTOR mRNA is a direct target of miR-100

**Fig. 6:**
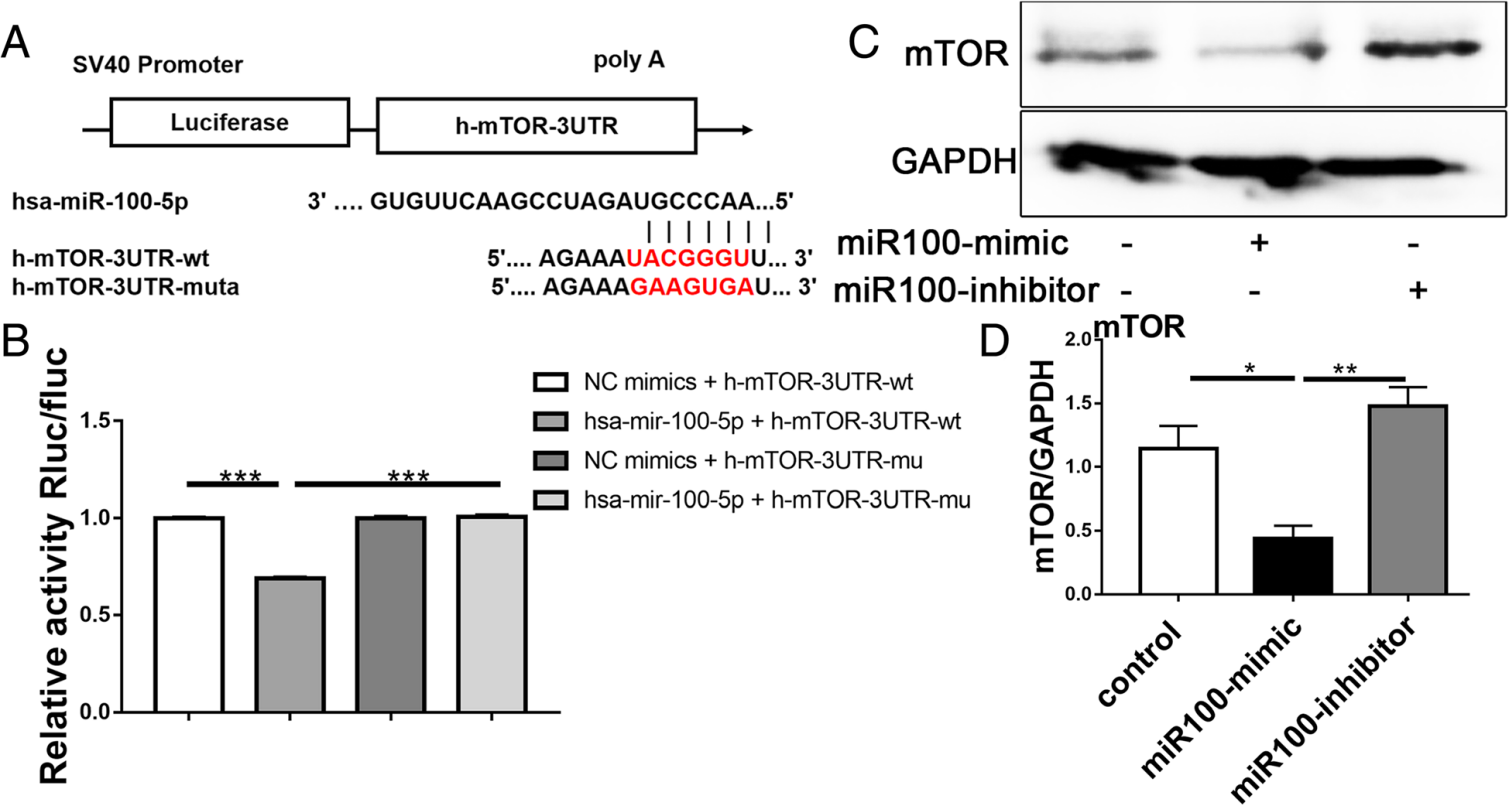
mTOR mRNA is a direct target of miR-100. **a** Sequences of the predicted miR-100-5p target sequences in the 3′UTR of *mTOR* mRNA and its mutant containing nucleotide substitutions in the 3′UTR. **b** The luciferase reporter assay was conducted to test whether the *mTOR* 3′UTR contains a binding site for miR-100. Luciferase assays show decreased reporter activity after co-transfection of the wild-type mTOR 3′UTR plasmid with miR-100 into 293T cells. **c** Protein expression of mTOR visualized by western blotting. GAPDH served as an internal control. **d** Protein semi-quantitative analysis of mTOR. Data are presented as mean ± SEM (*n* = 3). Statistical analysis was performed using one-way ANOVA followed by Tukey’s test; *P < 0.05, **P < 0.01, ***P < 0.001, ****P < 0.001

Luciferase reporter assay results confirmed that miR-100 overexpression decreased the luciferase activity in the wild-type group (*P* < 0.0001) but did not affect the mutant group (*P* < 0.0001; Fig. 6a, b). Figure 6c, d indicates that the protein level of mTOR was diminished by treatment with the miR-100 mimic, as indicated by the western blot results.

## Discussion

**Fig. 7:**
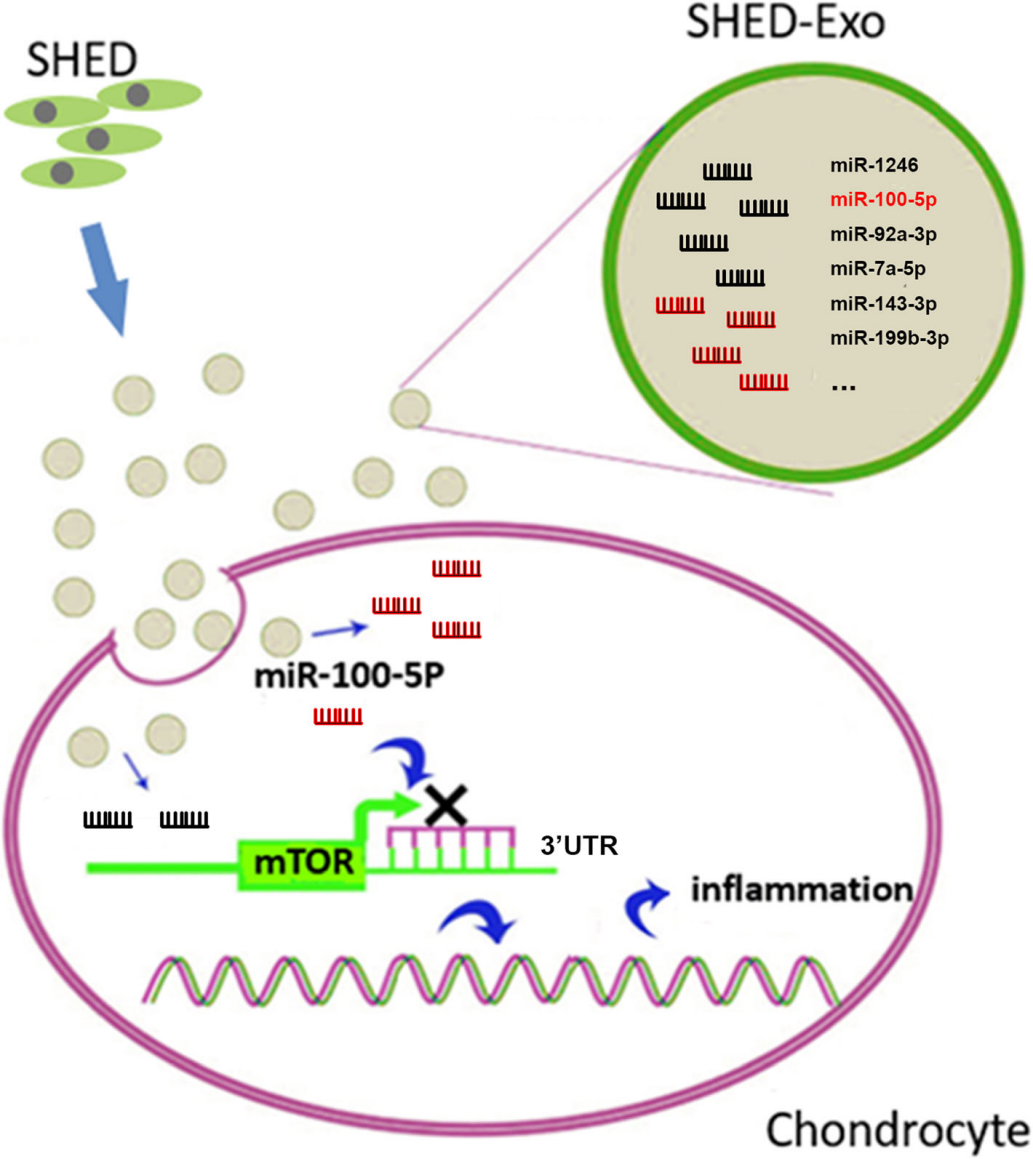
SHED-Exos as an anti-inflammatory agent. SHED-Exos, enriched with miR-100-5p, as the key anti-inflammatory agent in SHED secretion through the target mTOR in chondrocytes to suppress inflammation

This study revealed that SHED-Exos have anti-inflammatory effects in TMJOA. Furthermore, miR-100 was found to be enriched in SHED-Exos and served as an anti-inflammatory component of SHED-Exos (Fig. 7). In addition, this study demonstrated an important role of mTOR in TMJOA-associated inflammation: a downstream mediator of miR-100 action.

OA is the most common joint disease globally, but there is no standard treatment to prevent disease progression. Inflammation has been implicated in the pathogenesis of OA, suggesting that targeting inflammation in OA could be a promising therapeutic strategy [26]. Recently, treatment with MSC-derived exosomes has received much attention. Exosomes derived from MSCs are known play a critical part in the repair of tissue damage [27]. SHEDs, a type of MSCs, have advantages such as fewer ethical controversies, a readily accessible source, easy and minimally invasive collection, and retention of high stem cell potential such as cell proliferation, multipotency, and immunomodulatory functions compared to other MSCs [28]. Furthermore, accumulating evidence suggests that SHEDs exert curative action in various diseases including cord injury [29], bone defects [30], and ischemic brain injury [31]. Moreover, exosomes have functions similar to those of their parent cells and are safe and effective cell-free reagents. However, there was no study examining the effect of SHED-Exos on TMJOA. Based on these observations, we hypothesized that SHED-Exos may decrease the secretion of inflammatory factors in TMJOA. We were also eager to determine whether and how SHED-Exos downregulate inflammation in TMJOA.

First, to identify the presence of exosomes in a purified fraction, exosomes were confirmed by electron microscopy, NTA, and western blotting using exosome-specific biomarkers such as CD9, CD63, and TSG101 [32]. Next, to analyze the therapeutic potential of SHED-Exos in human TMJOA-associated inflammation, we conducted experiments revealing that both SHED-CM and SHED-Exos have the ability to ameliorate inflammatory changes in chondrocytes; each inhibited the expression of inflammatory cytokines including IL-6 and IL-8, and MMPs during IL-1β-induced inflammation. Previously, administration of SHED-Exos in a rat model of traumatic brain injury was shown to downregulate inflammatory cytokines, thereby supporting the notion that SHED-Exos have anti-inflammatory functions [15]. Recently, Zhang et al. reported that MSC exosomes alleviate temporomandibular joint osteoarthritis by attenuating inflammation and restoring matrix homeostasis. However, the underlying mechanism was unclear [33].

The anti-inflammatory effects of SHED-Exos in TMJOA were detected, but the exact mechanisms behind these effects have remained unknown. MiRNAs are an important component of exosomes and have been the focus of attention for many years. Many studies suggest that the presence of miRNAs in exosomes affects a variety of diseases including OA [34]. MiRNAs bind the 3′ untranslated regions (3′UTRs) of target mRNAs and suppress target protein synthesis [35]. To identify the specific SHED-derived exosomal miRNAs responsible for SHED-mediated anti-inflammatory effects, miRNA profile expression analysis was performed in the exosomes of human SHEDs. The results showed that exosomal miR-100 was significantly upregulated.

Several studies have demonstrated that miR-100 has a therapeutic potential in cancer [36]. However, the function of miRNA-100 associated with SHED-Exos in TMJOA-related inflammation is largely unknown. This study showed that an increase in miR-100 levels decreased the expression of inflammatory cytokines according to RT-qPCR. These results suggest that miR-100 plays an important role in the regulation of SHED-Exos. Moreover, Pankratz et al. recently reported that miR-100 can suppress chronic vascular inflammation, in line with our results on the effects of miR-100 on TMJOA-associated inflammation [37]. The data suggest that miR-100 is required for SHED-Exos to exert anti-inflammatory effects on TMJOA.

Next, we investigated the molecular mechanisms underlying the regulation of miR-100 expression. Functional enrichment analysis revealed that PI3K/AKT, MAPK, TNF-α, and estrogen signaling pathways are all associated with inflammation. The PI3K/AKT/mTOR pathway is believed to be related to autophagy and inflammation [38, 39]. In addition, Xue et al. have found that inhibition of PI3K/AKT/mTOR signaling attenuates the inflammatory response in rats with OA [40]. Furthermore, mTOR is closely related to proinflammatory signaling cascades of the MAPK [41], TNF-α [42], and estrogen pathways [43]. As the *mTOR* gene participates in the PI3K/AKT, MAPK, TNF-α, and estrogen signaling pathways, we focused on this gene. mTOR is the central signal integrator for nutrients and energy and performs critical functions in cell growth and division [44]. Several studies suggest that miR-100 directly targets the *mTOR* mRNA 3′UTR and represses mTOR expression in esophageal squamous cell carcinoma and chronic vascular inflammation [37, 45]. However, the mechanism underlying the involvement of miR-100 in TMJOA-associated inflammation has not been clarified yet. Therefore, we determined whether mTOR is regulated by miR-100 in TMJOA-associated inflammation. In our study, bioinformatics analysis revealed that *mTOR* mRNA contains a potential miR-100-5p-binding site. Western blot and RT-qPCR results confirmed that mTOR is upregulated by IL-1β but is downregulated by SHED-Exos and miR-100. Conversely, mTOR was upregulated by suppression of miR-100-5p expression. In addition, rapamycin, an allosteric inhibitor of the mTOR pathway, was used to inhibit mTOR. The data showed that after rapamycin treatment, miR-100 was overexpressed and proinflammatory cytokines were downregulated [46]. Moreover, luciferase reporter assay results further confirmed that *mTOR* mRNA is a direct target of miR-100. These data strongly indicate that the inflammation-suppressive function of miR-100 is mediated by attenuation of mTOR expression; this finding may have major implications for the mTOR pathway in TMJOA-associated inflammation. Herein, we report for the first time that SHED-Exos with miR-100-5p may participate in the maintenance of TMJ cartilage through mTOR. However, further studies are warranted to focus on the specific mechanisms that govern this process.

Further, genes in this KEGG network were significantly enriched in many other pathways, such as Ras signaling, FoxO signaling, and chemokine signaling pathways. Grabiec et al. found that reduced FoxO1 expression is required to promote the survival of fibroblast-like synoviocytes in rheumatoid arthritis [47]. Kono et al. reported that RasGRP-4, an intracellular signaling protein, is aberrantly expressed in fibroblast-like synoviocytes and helps regulate their growth; making it a candidate target for reducing proliferative synovitis and joint destruction [48]. In addition, chemokines and their receptors are found to play a significant role in arthritis processes [49]. All these pathways are related to osteoarthrosis, but the mechanisms associated with SHED need to be explored and validated further.

## Conclusions

In conclusion, our results suggest that miR-100, as a SHED-Exo-enriched miRNA, suppresses inflammation via repression of mammalian target of rapamycin (mTOR). We also demonstrated the therapeutic potential of novel anti-inflammatory SHED-Exos against inflammation in TMJOA. As it is relatively convenient and feasible to obtain SHED-Exos, and considering their low immunogenicity, our findings may help develop a novel potential therapeutic strategy against human TMJ inflammation.

## Data Availability

The datasets used and analyzed during the current study are available from the corresponding author on reasonable request.

## Abbreviations

ADAMTS5: Disintegrin and metalloproteinase with thrombospondin motifs 5
CM: Conditioned medium
Exos: Exosomes
GAPDH: Glyceraldehyde 3 phosphate dehydrogenase
IL-6: Interleukin-6
IL-8: Interleukin-8
miR-100: MicroRNA-100
miRNA: MicroRNA
MMP-9: Matrix metalloproteinase-9
MMP1: Matrix metalloproteinase-1
MMP13: Matrix metalloproteinase-13
MMP3: Matrix metalloproteinase-3
MSCs: Mesenchymal stem cells
mTOR: Mammalian target of rapamycin
NTA: Nanoparticle tracking analysis
OA: Osteoarthritis
RT-qPCR: Reverse transcription-quantitative polymerase chain reaction
SHEDs: Stem cells from human exfoliated deciduous teeth
TEM: Transmission electron microscopy
TMJ: Temporomandibular joint
